# Is a Ketogenic Diet Superior to a High-Fat, High-Cholesterol Diet Regarding Testicular Function and Spermatogenesis?

**DOI:** 10.3389/fnut.2022.805794

**Published:** 2022-02-10

**Authors:** Chin-Yu Liu, Ting-Chia Chang, Shyh-Hsiang Lin, Chih-Wei Tsao

**Affiliations:** ^1^Department of Nutritional Science, Fu Jen Catholic University, New Taipei City, Taiwan; ^2^School of Nutrition and Health Sciences, College of Nutrition, Taipei Medical University, Taipei, Taiwan; ^3^Master Program in Food Safety, College of Nutrition, Taipei Medical University, Taipei, Taiwan; ^4^Division of Urology, Department of Surgery, Tri-Service General Hospital, National Defense Medical Center, Taipei, Taiwan; ^5^Division of Experimental Surgery Center, Department of Surgery, Tri-Service General Hospital, National Defense Medical Center, Taipei, Taiwan

**Keywords:** high-fat and high-cholesterol diet, ketogenic diet, spermatogenesis, oxidative stress, apoptosis

## Abstract

The study aimed to determine effects of a ketogenic diet on metabolic dysfunction, testicular antioxidant capacity, apoptosis, inflammation, and spermatogenesis in a high-fat and high-cholesterol diet-induced obese mice model. Forty-two male C57BL/6 mice were fed either a normal diet (NC group) or a high-fat and high-cholesterol (HFC) diet (HFC group) for 16 weeks, and mice from the HFC group were later randomly divided into two groups: the first were maintained on the original HFC diet, and the second were fed a medium-chain triacylglycerol (MCT)-based ketogenic diet for 8 weeks (KD group). A poor semen quality was observed in the HFC group, but this was eliminated by the ketogenic diet. Both the HFC and KD groups exhibited enhanced apoptosis protein expressions in testis tissue, including caspase 3 and cleaved PARP, and higher inflammation protein expressions, including TNF-α and NF-κB. However, the KD group exhibited a statistically-significant reduction in lipid peroxidation and an increased glutathione peroxidase level as compared with the HFC group. The HFC diet induced obesity in mice, which developed body weight gain, abnormal relative organ weights, metabolic dysfunction, and liver injury. Overall, the results showed that a ketogenic diet attenuated oxidative stress and improved the semen quality reduced by the HFC diet.

## Introduction

Dietary composition nowadays has shifted from being staple-based toward containing higher levels of animal-source foods and vegetable oils. In addition, an increased total food consumption, higher added sugar levels, and greater intakes of fatty food and highly-processed food have established a calorically-dense dietary pattern, which has led to an obesogenic environment (1). Previous studies have shown that a higher BMI is associated with a lower live birth rate (2) and a poor semen quality (3). In addition, the amounts and types of fat intake (4), and even serum lipids, are associated with semen quality parameters (5), and evidence from human study has demonstrated high incidences of hypercholesterolemia and hypertriglyceridemia in infertile men (6). In addition to weight gain and metabolic change, obesity may cause oxidative stress, apoptosis and inflammation (7), which are involved in male infertility. Several studies have shown an association between obesity and high reactive oxygen species (ROS) production in sperm (8). Furthermore, semen of infertile men was found to have an increased ROS level, which was positively correlated with higher expressions of apoptotic mediators (9). Moreover, inflammation is associated with oxidative stress owing to ROS or free-radical overproduction during the inflammatory reaction (10).

The ketogenic diet is a dietary pattern incorporating low carbohydrate, high fat, and adequate protein, and was used to treat epilepsy in the 1920s. Since the 1960s, the ketogenic diet has been demonstrated to have significant effects in terms of weight loss (11). Previous studies showed that the ketogenic diet helped to lower body weight, serum lipids and blood sugar in obese patients (12). Medium-chain triacylglycerols (MCT) such as decanoic acid (C10:0), octanoic acid (C8:0), and hexanoic acid (C6:0) are saturated fatty acids with 6–10 carbons. Compared with long-chain triacylglycerols (LCT), MCT do not require chylomicron, and are rapidly absorbed through portal circulation and metabolized. MCT can enter the mitochondria without a carnitine shuttle for β-oxidation, and then produce excessive acetyl-CoA and increase the production of ketone bodies as another energy source for the brain, heart and kidneys, thus preventing fat mass accumulation (13). Results from human studies have indicated that MCT consumption could decrease body weight (14) and alter lipid profiles in overweight or obese individuals (15). There are few studies advocating the effects of a ketogenic diet or MCT in terms of improving fertility, and most have focused on female fertility (16). *In vitro* study has ever demonstrated that ketone bodies being utilized as an energy source for sperm movement (17). Moreover, weight loss in the overweight and obese is a viable strategy to decrease sperm DNA damage (18) and enhance semen quality (19).

Our recent study observed the effects of metformin on metabolic imbalance, antioxidant capacity, apoptosis, and reproductive function in male mice with high-fat and high-cholesterol diet-induced obesity, and demonstrated that it protected against the disrupted testicular histology and semen quality by increasing the testosterone concentration and enhancing anti-oxidative enzymes in the testes (20). The ketogenic diet is a popular weight loss strategy, and we performed this work in order to study whether this diet can reduce the impact of obesity and improve male reproductive function, and if possible to identify the related mechanisms.

## Methods

### Headings

This study was approved by the Institutional Animal Care and Use Committee of the Taipei Medical University (IACUC; ethical code number: LAC-2016-0358). The study was carried out in compliance with the ARRIVE guidelines, and all experiments were also performed in accordance with Taipei Medical University guidelines.

Eight-week-old male C57BL/6 mice (*n* = 42) were purchased from the National Laboratory Animal Center and housed in the laboratory animal center of Taipei Medical University (Taipei City, Taiwan). The animals were maintained at a room temperature of 23 ± 2°C with 12 h light/dark cycles (7 a.m./7 p.m.) under 50–60% relative humidity. After 2 weeks of adaption, the mice were randomly divided into two groups. Group 1 (normal control, NC, *n* = 10) received a normal diet (AIN-93G) with 70% of the total calories derived from carbohydrates, 20% from protein and 10% from fat, while Group 2 (high-fat and high-cholesterol diet, HFC, *n* = 32) were fed a high-fat diet plus 1.5% (w/w) cholesterol with 43% carbohydrates, 17% protein and 40% fat. The mice in Group 2 were randomly assigned to one of two groups, with 16 mice in each group: a high-fat and high-cholesterol diet (HFC) group and a ketogenic diet (KD) group after a period of 16-week induction of obesity. The HFC group were maintained on the original diet formula mentioned above, while the KD group were fed a ketogenic diet, in which the main resource of fat was changed from soybean oil to MCT oil, with 4% carbohydrates, 13% protein and 83% fat (Table 1). At the end of the 8-week experimental period, the mice were anesthetized with a mixture of Zoletil 50 (containing 25 mg/ml Tiletamine & Zolazepam; Virbac, Carros, France) and 2% Rompun (containing 20 mg/ml Xylazine; Bayer, Leverkusen, Germany), weighed and sacrificed. Blood samples were collected and centrifuged at 2,000 × g for 20 min, and serum was isolated and stored at −80°C until analysis. Samples of liver and testis tissue were either fixed in 10% formalin (J.T. Baker, NJ, USA) for histological evaluation or frozen in liquid nitrogen immediately and stored at −80°C for subsequent analysis. For measurement of sperm parameters, a needle containing 0.5 ml 1X phosphate-buffered saline (PBS, diluted from 10X PBS; Bioman, Taipei City, Taiwan) was used to flush out all spermatozoa from the vas deferens.

**Table 1:** Composition of experimental diets.

**Ingredients**	**NC**	**HFD**	**KD**
Corn starch	41.0	29.5	-
Dextrin	15.5	10.0	-
Sucrose	10.0	10.0	-
Cellulose	5.0	5.0	9.0
Casein	19.0	19.0	9.0
Soybean oil	4.0	20.0	-
MCT oil	-	-	77.0
Protein	-	-	8.47
Fat	-	-	53.90
Dietary fiber	-	-	19.01
Mineral mix	3.5	3.5	3.5
Vitamin mix	1.0	1.0	1.0
Choline	0.25	0.25	0.25
Cholesterol	-	1.5	1.5
L-Cysteine	0.18	0.18	0.18
TBHQ	0.25	0.0008	0.0008
Metformin	-	-	-
Energy (% of kcal)	NC	HFD	MCT
Carbohydrate	70	43	4
Fat	10	40	83
Protein	20	17	13

*MCT, Medium-chain triacylglycerols*.

### Histological Analysis

Formalin-fixed liver and testis tissues were analyzed at the Department of Pathology of Cardinal Tien Hospital (New Taipei City, Taiwan) after tissue processing. Tissues were cut, stained with Hematoxylin and Eosin (H&E) and evaluated using a light microscopy (DM1000; Leica, Wetzlar, Germany). Thickness of the germinal epithelium, mean seminiferous tubule diameter (MSTD) and testicular spermatogenesis were then measured using ImageJ software (1.50; National Institutes of Health, MD, USA). The testicular spermatogenesis was judged from the level of sperm maturation and scored from 1 to 10 according to Johnsen's testicular biopsy score system (21) to determine testicular sperm maturation in each group.

### Serum Analysis

Except serum triglycerides (TG) level was measured using a biochemical analyzer (DRI-CHEM 3500s; Fuji, Tokyo, Japan), other parameters including serum glucose (GLU), total cholesterol (TC), alanine aminotransferase (ALT) and aspartate aminotransferase (AST) were determined by hematologic analysis (ProCyte Dx; IDEXX, MA, USA). The serum insulin level was estimated using a commercial Elisa kit (Mercodia, 10-1247-01, NC, USA). The homeostasis model assessment of insulin resistance (HOMA-IR) was calculated as fasting blood glucose (nmol/L) multiplied by fasting serum insulin (μU/ml) divided by 22.5. The level of serum ketone bodies as β-hydroxybutyrate was measured using an Elisa kit (K632; Biovision, CA, USA) according to the manufacturer's instructions.

### Semen Quality Analysis

Sperm function parameters such as sperm motility, sperm count, and morphological abnormalities, following the WHO guidelines (22), were assessed using sperm samples. Samples from all groups were diluted and evaluated under a light microscope (E400; Nikon, Tokyo, Japan). At the same time, samples were incubated at 37°C for 15 min and estimated the sperm count using an automated cell counter (TC20; Bio-Rad, WA, USA). Sperm samples were then placed on a slide, air-dried at room temperature, fixed with methanol (Honeywell, WA, USA) for 5 min, stained with a mixture of Eosin Y (E4009; Sigma-Aldrich, MO, USA) and ethanol (Bioman), rinsed with 75% ethanol (Bioman) and dried. The slides were evaluated using a light microscope (DM1000; Leica) to estimate percentage of sperm of a normal morphology from a minimum of 100 spermatozoa. The remaining sperm samples were centrifuged, discarded the supernatant, collected sperm pellets, and stored at −80°C.

### Testicular Cholesterol and Sex Hormone Analyses

Frozen testis tissues were thawed, homogenized in an ice-cold radio immunoprecipitation assay (RIPA) lysis buffer (Thermo Fisher Scientific, Waltham, MA, USA), protease inhibitors and phosphatase inhibitors mixture, centrifuged at 14,000 × g (4°C) for 20 min and used the supernatant to measure the testicular concentrations of cholesterol, estradiol and testosterone with commercial ELISA kits according to the manufacturer's instructions (MBS164208; MyBioSource, San Diego, CA, USA; MBS261250; MyBioSource; Item No. 582701; Cayman, Ann Arbor, MI, USA).

### Western Blot Analysis

Testis tissues were processed as previously described and collected the supernatants. Briefly, determined the protein concentrations in each group using a detergent-compatible protein assay (5000002, Bio-Rad) and prepared equal quantities of protein (50 μg) samples. Samples were separated using sodium dodecyl sulfate (SDS)-polyacrylamide gels (Bioman), transferred onto polyvinylidene difluoride (PVDF) membranes (GE Healthcare, Freiburg, Germany), blocked with 5% (w/v) nonfat milk at room temperature for 1 h and incubated overnight at 4°C with primary antibodies. Primary antibodies including CYP11A1 (0.20 μg/ml; sc-202456), CYP17A1 (0.20 μg/ml; sc-66850), 3β-HSD (0.40 μg/ml; sc-28206), 17β-HSD (0.80 μg/mL; sc-135044), StAR (0.20 μg/ml; sc-25806), cytochrome C (0.20 μg/ml; sc-13156), PPAR⋎ (0.20 μg/ml; sc-7273), and IL6 (0.10 μg/ml; sc-57315) were purchased from Santa Cruz Biotechnology. The following primary antibodies were procured from Cell Signaling Technology (MA, USA) unless otherwise specified: Bax (0.20 μg/ml; 2772), caspase 9 (0.20 μg/ml; 9508), caspase 3 (0.40 μg/ml; 9662), cleaved-caspase 3 (0.80 μg/ml; 9664), PARP (0.20 μg/ml; 3542), caspase 8 (0.50 μg/ml; 59607; GeneTex, Texas, USA), Bcl-xl (0.40 μg/ml; ab32370; Abcam, MA, USA), TNF-α (0.25 μg/ml; ab1793; Abcam), NF-κB (0.20 μg/ml; E381; Abcam), and β-actin (0.02 μg/ml; A5316; Sigma, MO, USA).

The membranes were then washed with TBST (diluted from 10X TBST; Omicsbio, Taipei City, Taiwan), incubated with secondary antibodies Goat anti-mouse IgG-HRP (0.04 μg/ml, sc-2005; Santa Cruz Biotechnology, CA, USA) or Goat anti-rabbit IgG-HRP (0.04 μg/ml, sc-2054; Santa Cruz Biotechnology) for 1 h at room temperature., soaked in a chemiluminescent detection reagent (Omicsbio) for 1 min, and observed using a Chemiluminescent Imaging and Analysis System (MiniChemi TM 610; Sage Creation Science, Beijing, China). The protein level of β-actin was used for normalization, and the relative expression levels of target proteins were determined using ImageJ software.

### Testicular Antioxidants and MDA Levels

The activities of testicular antioxidants, superoxide dismutase (SOD), catalase (CAT) and glutathione peroxidase (GPx), and the MDA levels were measured using commercial Elisa kits (Cayman, #706002; Cayman, #707002; Cayman, #703102; Cayman, #10009055).

### Statistical Analysis

Data were expressed as means ± standard deviation (SD) and analyzed using SAS software (9.4; SAS Institute Inc., NC, USA). After ensuring the homoscedasticity of each group, one-way analysis of variance (ANOVA) with *post-hoc* comparisons using the least significant difference (LSD) test were used to compare differences among the three groups. A *p*-value lower than 0.05 (*P* < 0.05) was defined as significant.

The above-described methods of experimental analysis referenced previous published studies of our study group (20, 23).

## Results

### Body Weight and Biochemical Parameters

**Figure 1:**
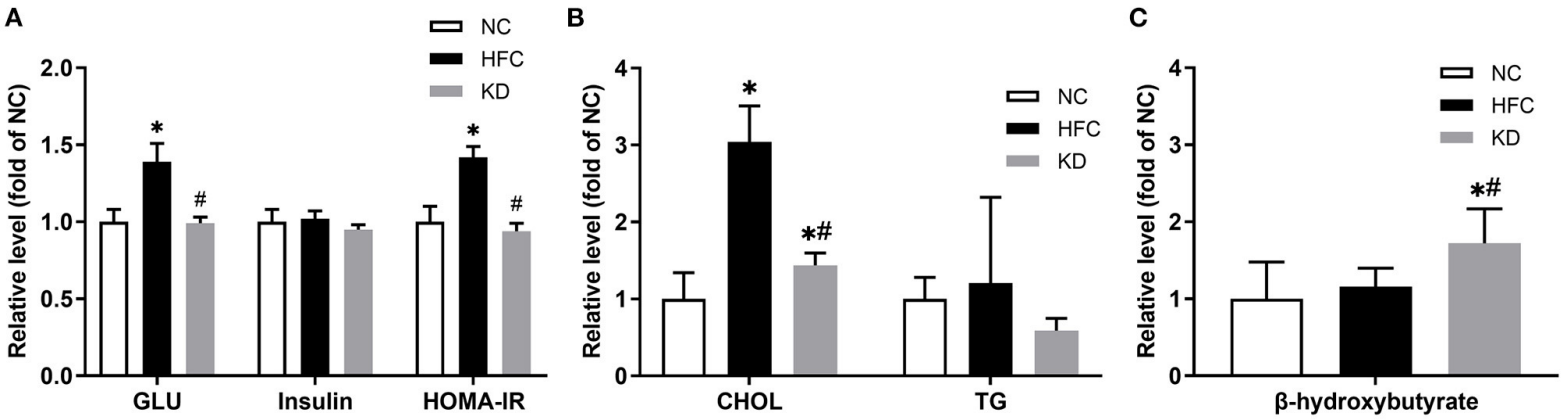
Effects of a high-fat and high-cholesterol diet and a ketogenic diet on **(A)** serum glucose (GLU), insulin and HOMA-IR, **(B)** serum total cholesterol (CHOL) and triglycerides (TG), and **(C)** serum β-hydroxybutyrate level in male mice. Data are expressed as the mean ± SD. *p < 0.05, compared with the NC group; ^#^*p* < 0.05, compared with the HFC group. NC, normal diet; HFC, high-fat and high-cholesterol diet; KD, HFC diet changed to MCT-based ketogenic diet.

The food intake amount of normal diet (NC) group, high-fat and high-cholesterol (HFC) diet group and ketogenic diet (KD) group were 4.1 ± 0.2 g/day/mouse, 3.3 ± 0.2 g/day/mouse and 3.1 ± 0.2 g/day/mouse. All mice of three groups had no water limitation. Mice fed a HFC diet showed a significant increase in body weight as compared with the NC group and the KD group (NC = 32.87 ± 2.73 g, HFC = 40.91 ± 4.21 g, KD = 31.21 ± 1.73 g). There was no significant difference in the mean kidney weight among the three groups, but the kidney to body weight ratio in the HFC group was markedly decreased. At the end of the 24-week experimental period, the biochemical parameters were measured. The HFC group had a higher serum glucose concentration and a higher HOMA-IR than the NC group (serum glucose: HFC = 555.50 ± 48.37 mg/dL, NC = 400.75 ± 32.90 mg/dL; HOMA-IR: HFC = 71.25 ± 3.45, NC = 50.36 ± 5.15); however, the ketogenic diet attenuated the HFC-induced elevated blood sugar and insulin resistance as measured by HOMA-IR (395.00 ± 15.45 mg/dL, 47.42 ± 2.53). In contrast to the NC group, the total cholesterol (TC) level was significantly increased in the HFC and KD groups (NC = 52.50 ± 17.78 mg/dL, HFC = 159.44 ± 24.56 mg/dL, KD = 75.78 ± 8.18 mg/dL; *p* < 0.05), but the serum TC in the KD group was significantly lower than that in the HFC group. Significant differences were not observed in the serum insulin and TG levels among the three groups. To evaluate blood ketone bodies, the β-hydroxybutyrate level was measured. Following an 8-week ketogenic diet, the mice in the KD group showed a marked increase in serum β-hydroxybutyrate as compared with the HFC and control mice (KD = 5.52 ± 1.43 nmol/μl, HFC = 3.70 ± 0.78 nmol/μl, NC = 3.20 ± 1.54 nmol/μl; *P* < 0.05, Figure 1).

### Liver Sections and Associated Parameters

**Figure 2:**
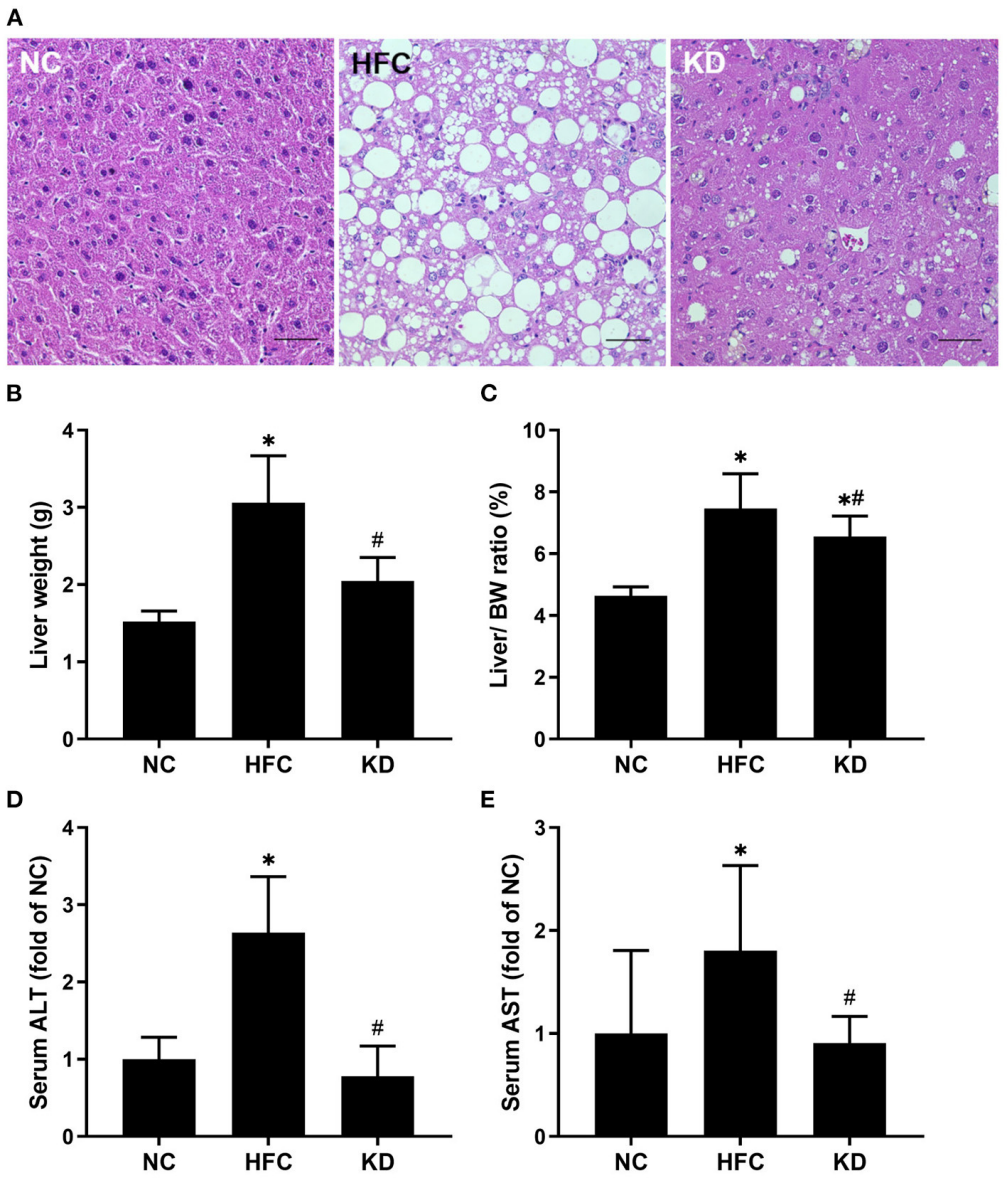
Effects of a high-fat and high-cholesterol diet and a ketogenic diet on **(A)** hepatic histology, **(B)** liver weight, **(C)** liver to body weight ratio, **(D)** serum ALT level, and **(E)** serum AST level in male mice. Hepatic sections were stained with hematoxylin and eosin. Data are expressed as the mean ± SD. *p < 0.05, compared with the NC group; ^#^*p* < 0.05, compared with the HFC group. NC, normal diet; HFC, high-fat and high-cholesterol diet; KD, HFC diet changed to MCT-based ketogenic diet. Scale bar: 50 μm.

Both the mean liver weight and the liver to body weight ratio were higher in the mice fed the HFC diet and the ketogenic diet as compared with the NC mice, but lower in the mice fed a ketogenic diet as compared with the HFC group. In addition, histological examination of sections of liver tissue illustrated that the HFC diet resulted in hepatic steatosis and enlarged lipid droplets, but the ketogenic diet decreased the amount and size of lipid droplets inside the liver tissue. The mice fed a normal diet had a normal liver histological structure with no presentation of steatosis. With regards to indicators of liver damage, the levels of ALT and AST were notably higher in the mice fed the HFC diet, whereas the ketogenic diet suppressed the HFC-induced increases in serum ALT and AST levels (Figure 2).

### Reproductive Organ Weights and Semen Quality

**Figure 3:**
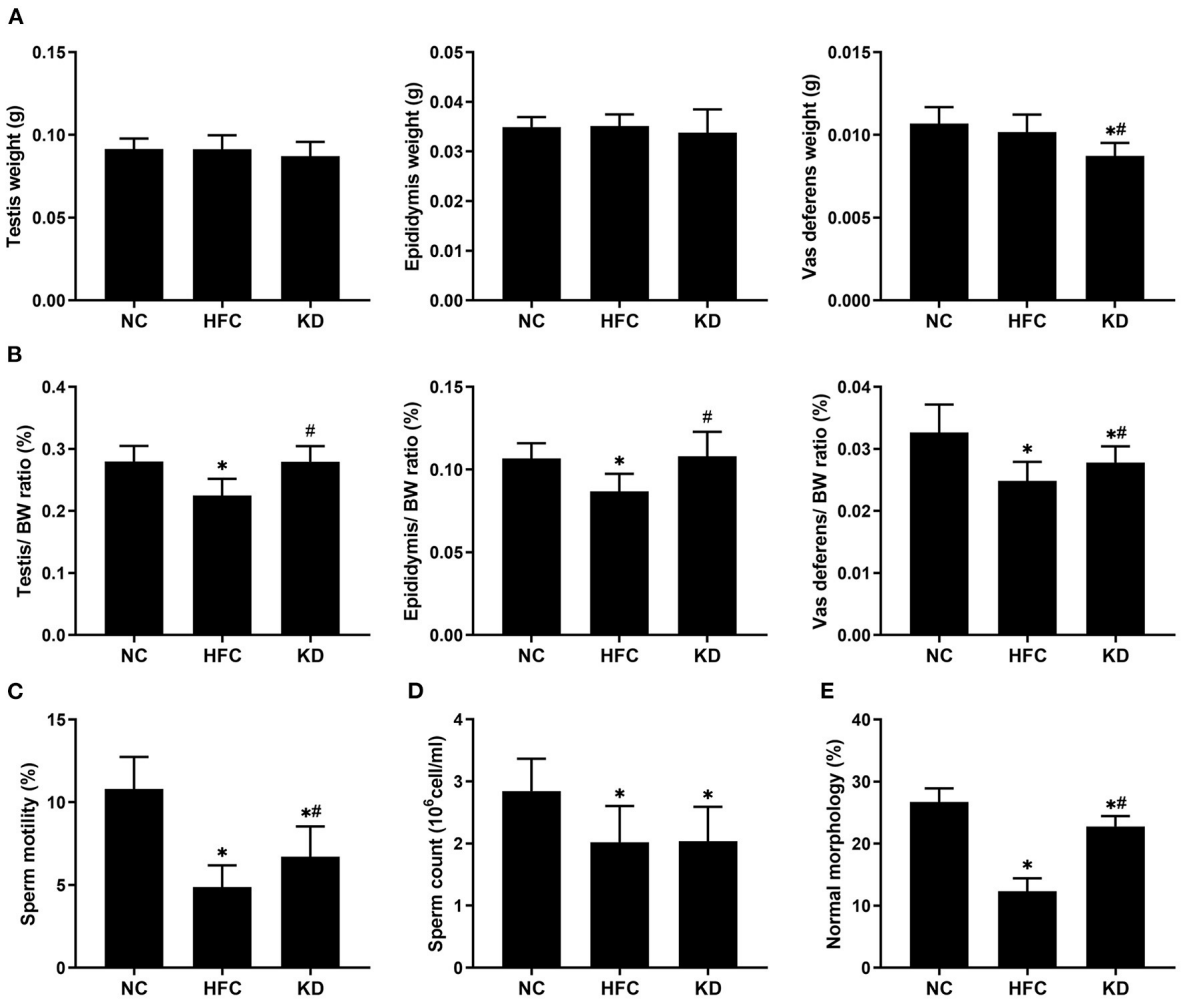
Effects of a high-fat and high-cholesterol diet and a ketogenic diet on **(A)** the weights of the testis, epididymis, and vas deferens, **(B)** the relative weights of the testis, epididymis, and vas deferens, **(C)** sperm motility, **(D)** sperm count, and **(E)** percentage of sperm of a normal morphology in male mice. *p < 0.05, compared with the NC group; ^#^*p* < 0.05, compared with the HFC group. NC, normal diet; HFC, high-fat and high-cholesterol diet; KD, HFC diet changed to MCT-based ketogenic diet.

No significant differences were observed in the mean testis and epididymis weights among the three groups, but the mean vas deferens weight in the KD group was higher than the weights in the NC and HFC groups. However, the testis, epididymis and vas deferens weight to body weight ratios were remarkably decreased in the HFC mice, and were improved in the mice fed a ketogenic diet. All sperm parameters in the HFC group showed significant reductions in comparison with the NC group. However, sperm motility and the percentage of sperm of a normal morphology were notably higher in the KD group. Sperm count was unaffected by the ketogenic diet and showed no significant difference from the HFC group (Figure 3).

### Testicular Sections, Testicular Cholesterol, and Testosterone Levels

**Figure 4:**
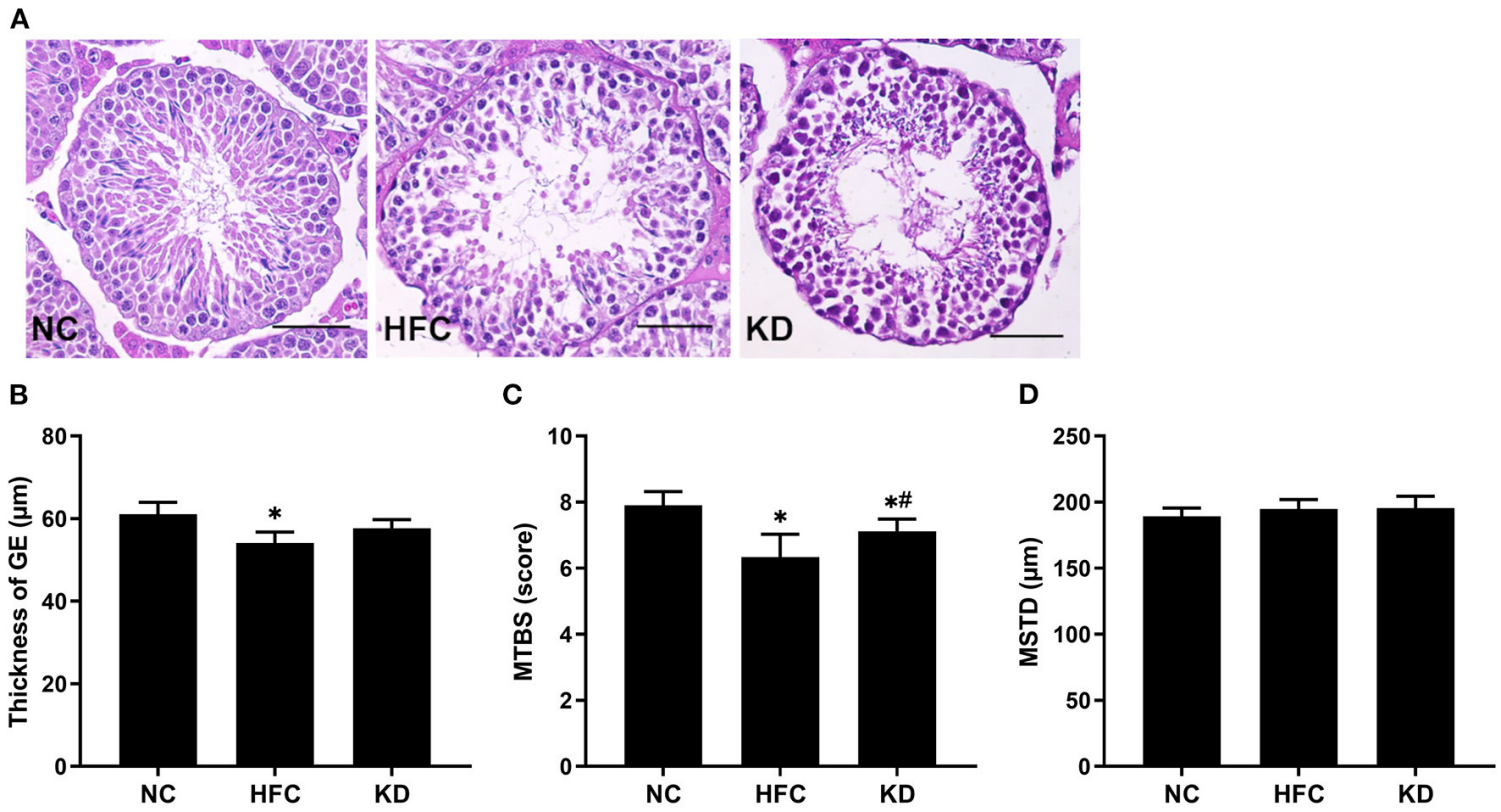
Effects of a high-fat and high-cholesterol diet and a ketogenic diet on **(A)** testicular histology, **(B)** thickness of the germinal epithelium (GE), **(C)** mean testicular biopsy score (MTBS), and **(D)** mean seminiferous tubule diameter (MSTD) in male mice. Testicular sections were stained with hematoxylin and eosin. *p < 0.05, compared with the NC group; ^#^*p* < 0.05, compared with the HFC group. NC, normal diet; HFC, high-fat and high-cholesterol diet; KD, HFC diet changed to MCT-based ketogenic diet. Scale bar, 50 μm.

The testosterone level was reduced and the cholesterol level elevated in the mice fed a HFC diet. In addition, the mice fed a ketogenic diet exhibited a decreasing trend in cholesterol concentration and an increasing trend in testosterone concentration, but these trends did not reach statistical significance. However, the estradiol level was statistically significantly elevated in the HFC and KD groups as compared with the NC group (Table 2). As mentioned above, a decreased testicular testosterone concentration was observed in the HFC group, suggesting that spermatogenesis was negatively affected, which may be related to abnormal testosterone biosynthesis. Sperm maturation was evaluated by H&E staining, the slides being observed under an electron microscope. Cross-sections from the testes in the NC group indicated a normal testicular morphology. Histological examination of the testes revealed that the HFC mice were characterized by a thinner germinal epithelium, desquamation of immature germ cells, and fewer mature spermatozoa as compared with the mice fed a normal diet. Following scoring according to Johnson's scoring method, the NC group had a higher mean testicular biopsy score (MTBS) than the HFC and KD groups, whereas the ketogenic diet improved sperm maturation. There were no significant differences in the mean somniferous tubule diameter (MSTD) among the three groups (Figure 4).

**Table 2:** Effects of a high fat and high cholesterol diet and a ketogenic diet on testicular cholesterol, testosterone, and estradiol levels in male mice.

	**Group**		
	**NC**	**HFC**	**KD**
Cholesterol (μM)	475.24 ± 7.53	490.89 ± 6.20^*^	484.36 ± 6.07
Testosterone (ng/ml)	194.37 ± 56.81	115.57 ± 36.08^*^	144.04 ± 56.65
Estradiol (ng/ml)	4.73 ± 0.75	7.16 ± 0.78^*^	7.05 ± 0.93^*^

*NC, normal diet; HFC, high fat and high cholesterol diet; KD, HFC changed to medium-chain triacylglycerols (MCT)-based ketogenic diet. Data are expressed as the mean ± SD*.

* *p < 0.05, compared with the NC group according to one-way ANOVA followed by LSD post-hoc comparison*.

### Protein Expressions and Activities of Testosterone Biosynthesis-Related Enzymes

**Figure 5:**
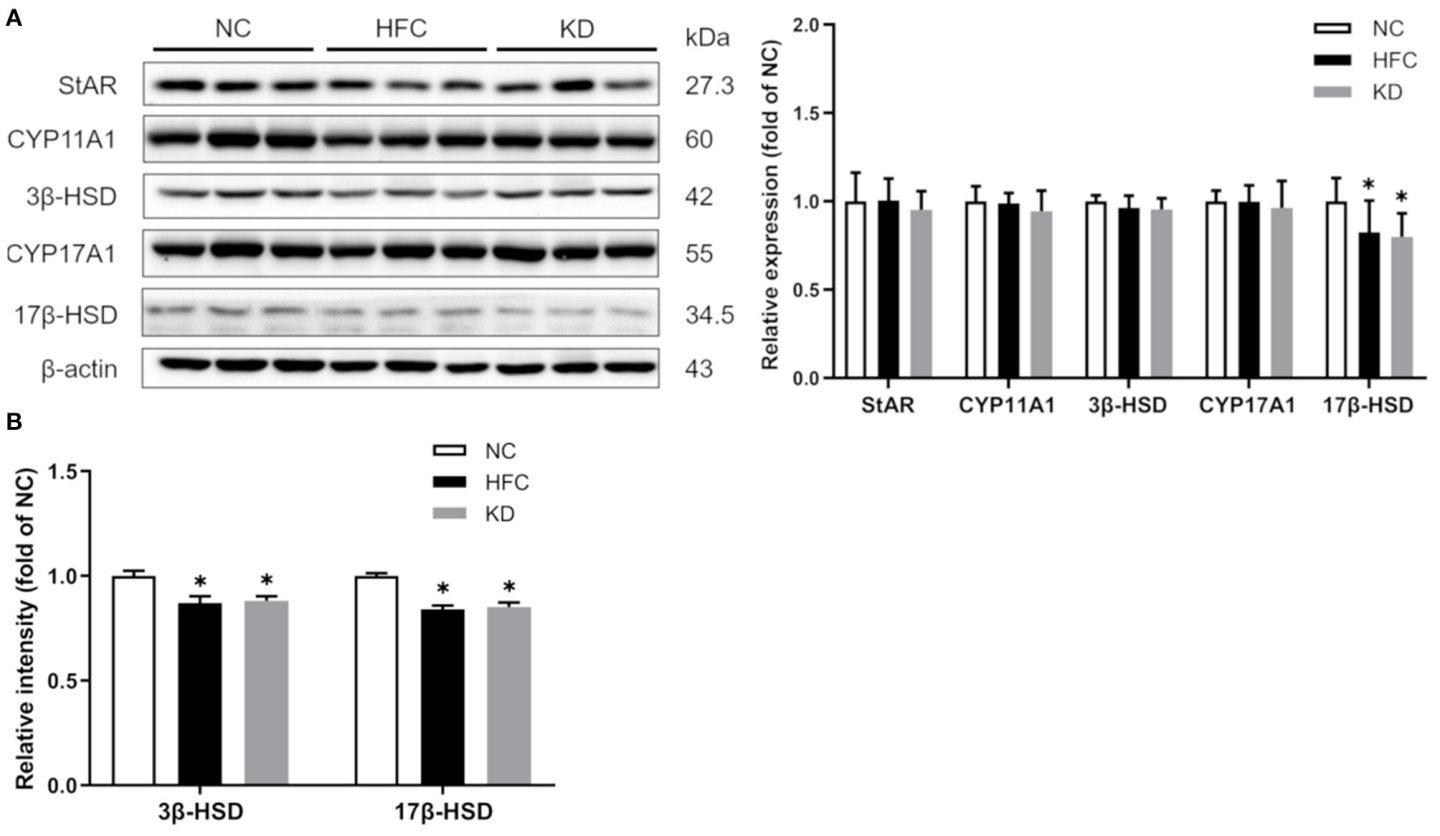
Effects of a high-fat and high-cholesterol diet and a ketogenic diet on **(A)** testosterone biosynthesis regulators (StAR, CYP11A1, 3β-HSD, CYP17A1, and 17 β-HSD) in male mice. Protein expressions in each group were measured using western blot analysis, and β-actin was used for normalization. **(B)** Activities of hydroxysteroid dehydrogenases (HSDs) were estimated using Elisa kits. Data are expressed as the mean ± SD. *p < 0.05, compared with the NC group; ^#^*p* < 0.05, compared with the HFC group. NC, normal diet; HFC, high-fat and high-cholesterol diet; KD, HFC diet changed to MCT-based ketogenic diet.

Moreover, testosterone synthesis-related proteins were detected by Western blotting, and the results showed that in comparison with the NC group, the testicular protein expression of 17β-HSD was significantly reduced in the HFC and KD groups. No remarkable differences were found in StAR, CYP11A1, 3β-HSD or CYP17A1 among the three groups. Owing to decreased protein expressions, the enzyme activities of HSDs were measured, and the HFC group mice exhibited lower activities of 17β-HSD than the control mice (Figure 5).

### Oxidative Stress-Associated Parameters

**Figure 6:**
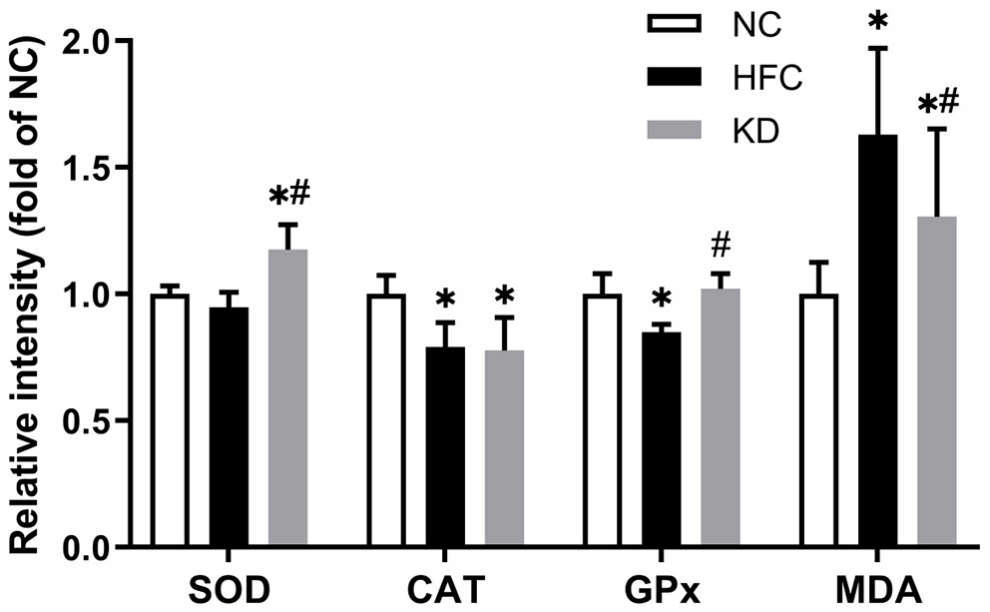
Effects of a high-fat and high-cholesterol diet and a ketogenic diet on testicular antioxidants [superoxide dismutase (SOD), catalase (CAT) and glutathione peroxidase (GPx) activity] and MDA content in male mice. Data are expressed as the mean ± SD. *p < 0.05, compared with the NC group; ^#^*p* < 0.05, compared with the HFC group. NC, normal diet; HFC, high-fat and high-cholesterol diet; KD, HFC diet changed to MCT-based ketogenic diet.

Testicular antioxidant enzymes including superoxide dismutase (SOD), catalase (CAT) and glutathione peroxidase (GPx) were examined. The SOD activity was significantly higher in the KD group than in the control and HFC groups. The CAT and GPX activities were notably decreased in the HFC group as compared with the NC group, and only the GPX activity was restored after changing to a ketogenic diet, while no significant differences were observed between the NC and KD groups. Additionally, the level of testicular lipid peroxidation was assessed by measuring the level of malondialdehyde (MDA), and the HFC diet led to an increase in MDA content as compared with a normal diet, whereas the ketogenic diet markedly decreased the MDA content (Figure 6).

### Protein Expressions of Apoptotic and Inflammatory Markers

**Figure 7:**
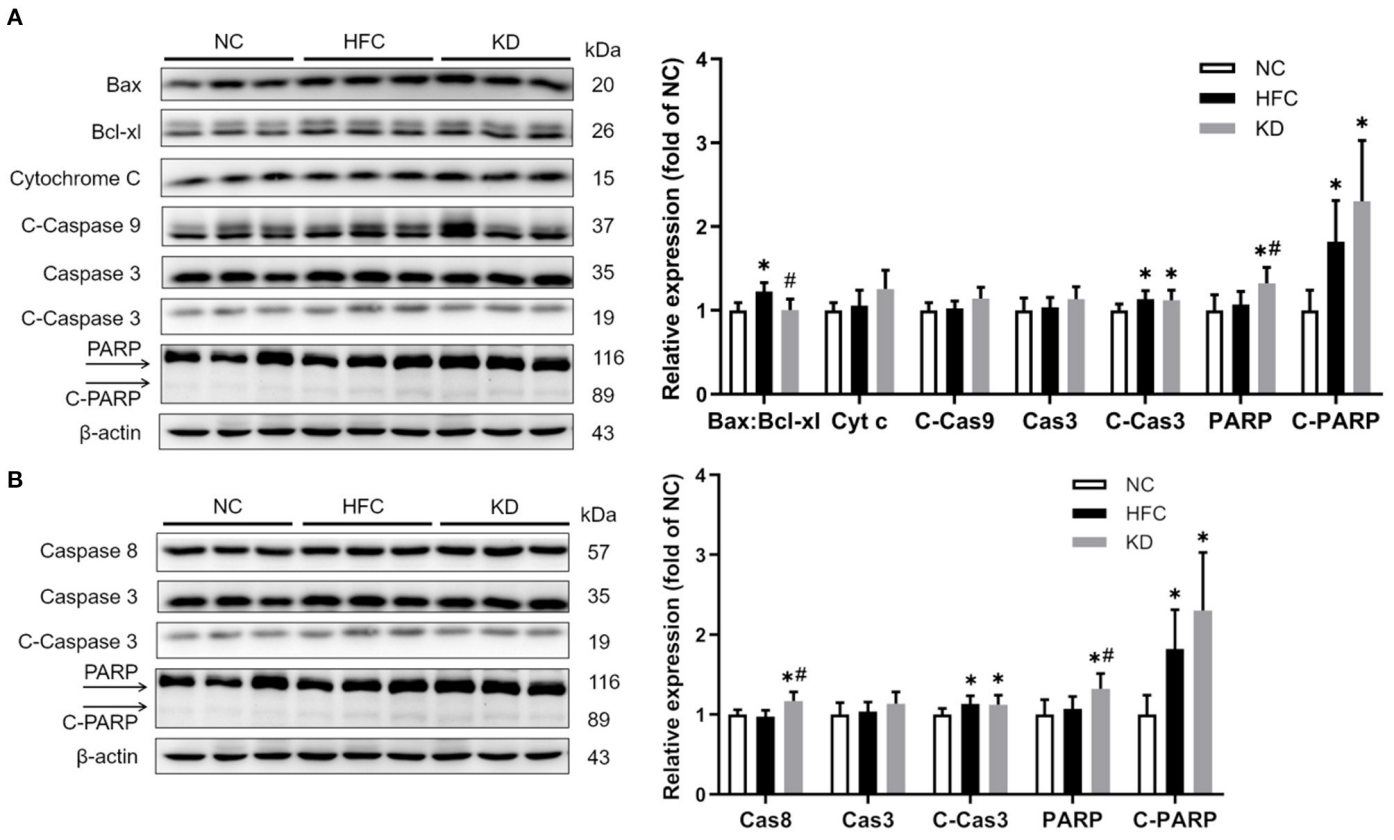
Effects of a high-fat and high-cholesterol diet and a ketogenic diet on testicular **(A)** intrinsic and **(B)** extrinsic apoptosis pathway regulators, including the ratio of Bax:Bcl-xl and the levels of cytochrome c (Cyt c), caspase 3 (Cas3), cleaved-caspase 3 (C-Cas3), caspase 8 (Cas8), cleaved-caspase 9 (C-Cas9), PARP, and cleaved-PARP (C-PARP) in male mice. Protein expressions in each group were measured using western blot analysis, and β-actin was used for normalization. Data are expressed as the mean ± SD. *p < 0.05, compared with the NC group; ^#^*p* < 0.05, compared with the HFC group. NC, normal diet; HFC, high-fat and high-cholesterol diet; KD, HFC diet changed to MCT-based ketogenic diet.

**Figure 8:**
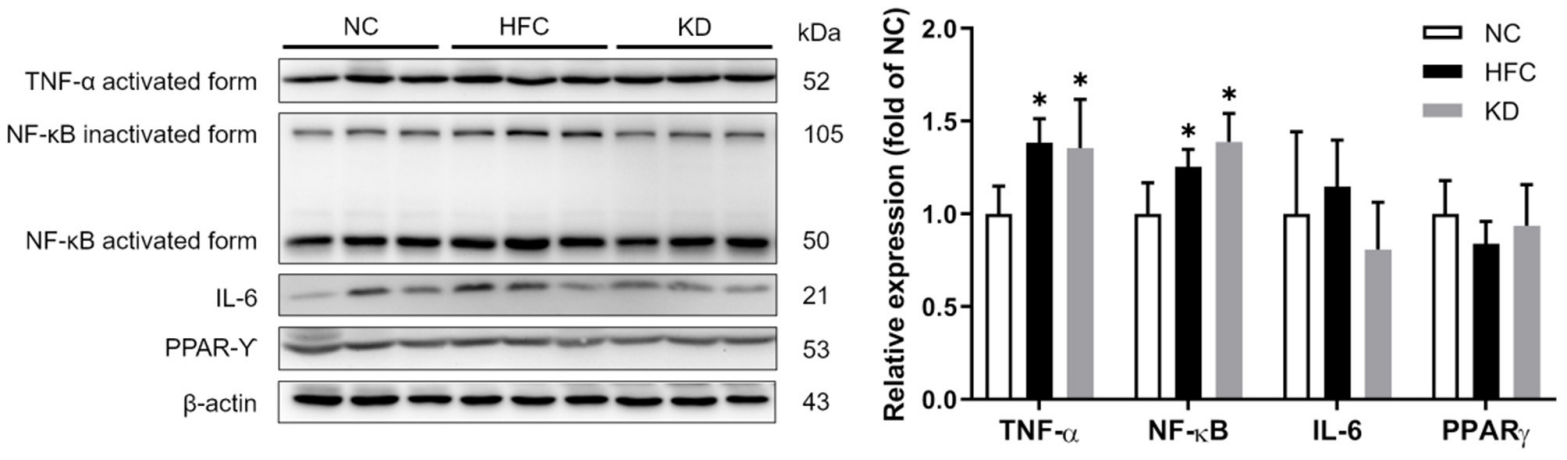
Effects of a high-fat and high-cholesterol diet and a ketogenic diet on testicular inflammation pathway regulators (TNF-α, NFκB, IL6, and PPAR⋎) in male mice. Protein expressions in each group were measured using western blot analysis, and β-actin was used for normalization. *p < 0.05, compared with the NC group; ^#^*p* < 0.05, compared with the HFC group. NC, normal diet; HFC, high-fat and high-cholesterol diet; KD, HFC diet changed to MCT-based ketogenic diet.

Western blotting revealed that the protein expressions of the cleaved forms of PARP and caspase 3 were significantly upregulated in the HFC group, and these changes were not eliminated in the KD group. In addition, the ratio of anti-apoptotic Bcl-xl to pro-apoptotic Bax was found to be higher in the HFC mice, while the levels of caspase 8 and PARP were significantly increased in the KD mice (Figure 7). Inflammation-related mediators including TNF-α, NF-κB, IL-6, and PPAR-⋎ were assessed, and the results showed increased protein levels of testicular adipokine, TNF-α and NF-κB in the HFC group as compared with the control mice. The elevated adipokine levels of TNF-α and NF-κB were not obliterated in the group fed a ketogenic diet. There were no significant differences in the expressions of IL-6 and PPAR⋎ among the NC, HFC, and KD groups (Figure 8).

## Discussion

In the diet-induced obesity mouse model employed in this study, the animals developed body weight gain, abnormal relative organ weights, metabolic dysfunction, and liver injury. The weight imbalance in the mice fed a high-fat and high-cholesterol (HFC) diet was later significantly reversed by an MCT-based ketogenic diet. These results were similar to those of other studies that revealed the efficacy of the ketogenic diet, and several mechanisms have been discussed (11), which include appetite reduction (24), increased lipolysis with decreased lipogenesis (25), greater satiety, and a thermic effect due to protein consumption (26). The biochemical analysis results showed that the ketogenic diet suppressed the higher blood sugar and total cholesterol in addition to insulin resistance due to the HFC diet. Dashti et al. (27) declared significant decreases in blood glucose, total cholesterol, LDL and TG in obese diabetic subjects following a ketogenic diet. Similarly, an animal study demonstrated that replacement of lard by MCT in a high-fat diet ameliorated changes to fasting blood glucose, insulin, and insulin resistance (28). Interestingly, no significant differences were observed in the mean concentration of serum TG in this study. Tamada et al. (29) reported that the HFC diet may inhibit the catabolism and synthesis of TG by downregulating the expressions of related enzymes. With regards to hepatic histology and function, a high fat and cholesterol intake has often been established as a model of progression of non-alcoholic fatty liver disease (NAFLD) to steatohepatitis and fibrosis (30). The HFC group mice exhibited lipid accumulation and hepatic steatosis in liver tissue, as well as higher AST and ALT levels, which may lead to upregulation of intestinal cholesterol transporters and increased cholesterol absorption; this then induces elevated hepatic endoplasmic reticulum stress and causes cell death (31). A ketogenic diet improved the hepatic sections and functions, consistent with a randomized controlled human study, which showed that education regarding a low carbohydrate diet significantly lowered liver enzyme levels as compared with NAFLD patients following a low-fat diet (32).

Various studies have revealed an association between obesity and male reproductive function. An observational study demonstrated that underweight and overweight individuals have a lower semen quality, in terms of total sperm number and total motile sperm count (33). Adverse effects of a high fat (34) or high cholesterol intake (35) on the structure and function of the testes have been reported. In this study, HFC consumption induced pathological damage in testicular tissue and impaired spermatogenesis in terms of reduction of the germinal epithelium thickness, fewer mature spermatozoa, and a poor semen quality. However, the sperm motility, percentage of sperm of a normal morphology and spermatogenic cell maturation were enhanced by the ketogenic diet. Leptin receptor-deficient db/db mice developed characteristics of hyperphagia, an extreme blood sugar level, high lipid levels, severe obesity, and infertility, whereas an MCT diet in that animal model improved spermatogenesis and spermiogenesis (36). Furthermore, ketone bodies can serve as alternative fuel for sperm and motivate sperm motility (18).

The HFC-fed mice had higher testicular cholesterol concentrations; however, the testosterone level was decreased and the estradiol level increased. Several studies have indicated that hypercholesterolemia is associated with male infertility, including testicular and sperm dysfunction (37). Moreover, a lower testosterone concentration may be due to the expression of aromatase in adipose tissue, which converts testosterone to estradiol. Another possible explanation is that the hypothalamic-pituitary-gonadal axis is interfered with under high testicular cholesterol and estradiol levels (38). The ketogenic diet slightly decreased the cholesterol level and increased the testosterone level, while there were no significant differences between the HFC and KD groups. We also analyzed the expressions of testosterone synthesis-related enzymes, and the results showed that the protein expression and enzyme activity of 17β-HSD were greatly reduced in both the HFC group and the KD group, suggesting that the conversion of androstenedione to testosterone was blocked (39).

Excess oxidative stress resulting in overproduction of ROS and reductions in anti-oxidative enzymes has been demonstrated to be a possible mediator of male infertility (40). A high concentration of ROS easily causes oxidative damage owing to the abundance of polyunsaturated fatty acids in sperm membranes, and leads to lipid peroxidation, sperm DNA damage and apoptosis (41). Our results showed increased lipid peroxidation and a higher MDA content, along with a decreased testicular antioxidant defense system function in the HFC-fed mice, but the MCT-based ketogenic diet suppressed MDA production and motivated the activities of anti-oxidative enzymes. Previous studies showed that the MCT and ketogenic diet could reduce lipid peroxidation (42) and upregulate gene expressions of anti-oxidative enzymes through activating nuclear factor erythroid-derived 2 (NF-E2)-related factor 2 (Nrf2) (43). Excessive apoptosis (44) and inflammation (10) may lead to inhibition of sperm production and worsen semen quality. The HFC diet could induce apoptosis and inflammation reactions, as identified in several studies (31, 45); moreover, the ketogenic diet also has significant effects on apoptosis- and inflammation-related protein expressions. These findings were inconsistent with other studies (46, 47) demonstrating that the anti-inflammatory effects of a ketogenic diet may contribute to the recovery of neurologic function after spinal cord injury and provide both structural and functional neuroprotective effects in a multiple sclerosis murine model, suggesting that male testicular and reproductive tissue differs from neurologic organs, being too susceptible to oxidative stress to recover from the inflammation process. Herein further study should enroll the non-obese experimental group that consumed a ketogenic diet, to compare with other groups to clarify the detailed inflammation or anti-inflammatory mechanism.

## Conclusion

The results of this study demonstrated that a high-fat and high-cholesterol diet caused reduced hormone levels, decreased spermatogenesis and a poor semen quality through elevated oxidative stress, apoptosis, and inflammation. Even though there was no greater enhancement in testosterone levels, testicular apoptosis and inflammation, a ketogenic diet may improve spermatogenesis and sperm parameters via enhancing the anti-oxidant capacity and reducing lipid peroxidation. The results of this study demonstrated the damage caused by a fat-enriched diet to the male reproductive function and possible related pathways.

## Data Availability Statement

The original contributions presented in the study are included in the article/supplementary material, further inquiries can be directed to the corresponding author/s.

## Ethics Statement

The animal study was reviewed and approved by the Institutional Animal Care and Use Committee of the Taipei Medical University (IACUC; ethical code number: LAC-2016-0358).

## Author Contributions

C-YL conceived the study and wrote the manuscript. C-WT participated in the design of the study and helped draft the manuscript. T-CC participated in the statistical analysis and interpretation. S-HL participated in the coordination of the study design and helped draft the revised manuscript. All authors have read and approved the final manuscript.

## Funding

Our project was partially funded through research Grant A0110014 from Fu Jen Catholic University, Taiwan and Grant TSGH-D-111074 from Tri-Service General Hospital, Taiwan.

## Conflict of Interest

The authors declare that the research was conducted in the absence of any commercial or financial relationships that could be construed as a potential conflict of interest.

## Publisher's Note

All claims expressed in this article are solely those of the authors and do not necessarily represent those of their affiliated organizations, or those of the publisher, the editors and the reviewers. Any product that may be evaluated in this article, or claim that may be made by its manufacturer, is not guaranteed or endorsed by the publisher.
